# Molecular characterization of human adenovirus associated with acute respiratory infections in Cameroon from 2011 to 2014

**DOI:** 10.1186/s12985-018-1064-x

**Published:** 2018-10-03

**Authors:** Sebastien Kenmoe, Marie-Astrid Vernet, Jerôme Le Goff, Véronique Beng Penlap, Astrid Vabret, Richard Njouom

**Affiliations:** 1 Virology Department, “Centre Pasteur du Cameroun”, P.O.Box 1274, Yaounde, Cameroon; 2 Saint Louis Hospital, Paris, France; 3 Département de Biochimie, Université de Yaoundé 1, BP 812 Yaounde, Cameroon; 4 Normandie Université, 14032 Caen, France; 5 UNICAEN, UNIROUEN, GRAM, 14000 Caen, France; 6 University Hospital of Caen, Department of Virology, 14000 Caen, France

**Keywords:** Cameroon, Molecular characterization, Human adenovirus

## Abstract

**Background:**

Human adenoviruses (HAdVs) cause a wide range of diseases worldwide, including respiratory infections. Studies on HAdV molecular epidemiology are limited in Cameroon. The purpose of this study is to document the different types HAdV circulating in Cameroon in children with acute respiratory infections.

**Methods:**

Nasopharyngeal swabs were collected from 811 children under 15 years from 2011 to 2014. The HAdV detection was assessed by semi-quantitative generic PCR r-gene®. The HAdV-positive samples were typed by amplification and sequencing of partial hexon gene and a real-time PCR. Demographic data were collected and analyzed. The infection and hospitalization risk factors were assessed thought the Chi-square test.

**Results:**

A total of 137/220 HAdV-positive samples were amplified successfully. Six species of HAdV (*Mastadenovirus* A to F) were detected with B (108/220) and C (47/220) being the predominant strains. Hospitalization and age were significantly associated to HAdV-B and HAdV-C respectively. Phylogenetic analysis of HAdV-B3 virus (18) and B7 (5) shows a conserved and a significant temporal stability in relation to the reference sequence (99.1 to 100% of similarity).

**Conclusion:**

This study reported HAdV species and types detected in children with acute respiratory infections in Cameroon between September 2011 and July 2014. These results support further evaluation of the spatio-temporal circulation pattern of HAdV species and types in Cameroon.

## Background

Human adenoviruses (HAdVs) are one of the common pathogens found in acute respiratory infections worldwide. About 6–50% of respiratory infections are caused by HAdV in children [1–5]. This virus is also one of the most common causes of morbidity and mortality among children, particularly in developing countries. A study by McMorrow and colleagues shows that HAdV was detected in 6% of 834 deaths in patients with severe respiratory infections recorded across eight African countries [6]. HAdV is consisting of unenveloped capsid with icosahedral symmetry and diameter between 70 and 90 nm. This box surrounds the genome that is associated to 4 polypeptides (V, VII, X, and μ) and consisting of linear double-stranded DNA, non-segmented and ranging in size from 26 kb to 48 according to the gender. HAdVs are members of the family *Adenoviridae* and gender *Mastadenovirus*. Traditionally these viruses were classified by neutralization reactions, hemagglutination and hydrolysis with restriction enzymes in 51 serotypes and multiple types. To date, they are classified into 7 groups named species *Mastadenovirus* A to G with a total of 54 types officially recognized by the International Committee on Taxonomy of Viruses (ICTV). This classification is based on the partial (Hexon, penton base and fiber) or complete viral genome [7, 8]. New types of HAdV (HAdV-55 to 69) have recently been identified based on whole genome analysis. According to their tissue tropism, the various HAdV types are responsible of a wide range of human diseases. HAdVs species B, C, and, E are involved in upper and lower acute respiratory tract infections. Effective vaccine against HAdV strains is available only for types E (4) and B (7) [9]. To date, there is no antiviral treatment approved against HAdV infections. However, significant progress has been reported on cidofovir, a nucleotide analog that inhibits viral DNA polymerase [10]. The epidemiology, clinical presentation, pathogenesis, vaccination and treatment of HAdV infections depend on the viral type implicated. Thus, typing HAdV is therefore crucial for patient care and limiting the spread of infection [11]. Although HAdV has been studied in great detail for more than half century, data on circulating types in Cameroon remain completely unknown. HAdVs could have a particular epidemiology in African countries. High prevalence of HAdV have also been reported in African countries such as South Africa (31.4%) [12], Senegal (50%) [5], and Kenya (29.6%) [3] among patients of all ages with severe or mild acute respiratory infections. Similarly, a previously study on HAdV among Cameroonian hospitalized children showed that HAdV was the predominantly detected respiratory viruses (27.3%) [13]. In this study we perform molecular typing of HAdV strains detected in Cameroon between 2011 and 2014 and we also find hospitalization risk factors related to the different HAdV types isolated in Cameroon.

## Methods

### Study design

**Fig. 1:**
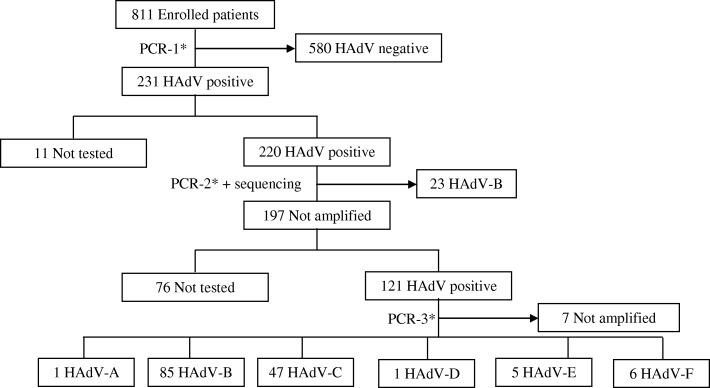
Flow chart of 811 patients with influenza-like illness enrolled in Cameroun, 2011–2014. *PCR-1: AdV/HBoV Respiratory Multi Well System r-geneTM (bioMérieux, Lyon, France), *PCR-2: PCR end point developed by Sriwanna et al. [15], *PCR3: In-house real-time PCR developed Leruez-Ville et al. and Watzinger et al. [16, 17]

This cross-sectional study was performed between September 2011 and July 2014. The samples were collected from outpatient or inpatient children at the pediatric service of the National Social Insurance hospital in Yaounde, Cameroon. Pediatric patients recruited met the case definition of WHO Influenza-Like illnesses (fever> 38 °C accompanied by cough or sore throat with symptoms dating the previous 5 days of inclusion). Overall 231 (28.4%) out of 811 tested samples were positive for HAdV (Fig. 1).

### Ethical approval

Ethical approval was obtained from the National Research Ethics Committee and the Ministry of Health of Cameroon for the main study (IMMI Project) associated with this work. This protocol was carried out in accordance with all the regulations establishing the protection of study participants. Informed written consent was acquired from the parents or guardians of all children involved in the study. A coding system was used for all participants to protect their confidential data. The caregivers of children were properly informed about the purpose of the study, the benefits and risks of participation in the study.

### Sampling, extraction of nucleic acids, detection, and typing of HAdV

Nasopharyngeal swabs were screened to detect respiratory viruses using r-gene commercial duplex AdV/HBoV r-gene™ (Respiratory Multi Well System r-gene™, BioMerieux, Lyon, France) as described elsewhere [14]. A total of 220 HAdV positive samples were used for further characterization by PCR carried out on hexon gene. To this end the test was carried out using primers published by Sriwanna et al. in 2013 [15]. Briefly, 5 μL of extracted DNA were added to a PCR mixture containing: 5 μL of 10X PCR buffer, 1.5 μL of MgCl_2_ at 50 mM, 1 μL of dNTP at 10 mM, 2.5 μL of W1 solution at 1%, 0.5 μL of Taq DNA polymerase (Invitrogen, USA), 29.5 μL of distilled water, 2.5 μL of the forward primer ADV_F2 (5’-TTY GCN ACC ATG CCC AAC AC-3 ‘), and 2.5 μL of the reverse primer AdV_R2 (GYY TCR AYG GCC ATG CGG TG) to a final volume of 50 μL. The thermal profile used consisted of initial denaturation at 94 °C for 3 min; followed by 40 cycles of 94 °C for 45 s, 50 °C for 30 s and 72 °C for one minute 30 s; and a final extension at 72 °C for 10 min. The primers amplify a partial hexon gene of 956 bp. Amplified hexon sequences were sequenced bidirectionally using the same PCR primers. The species of not amplified samples (121 cases) after research by end point PCR of the Hexon gene have been identified as HAdV-A, B, C, D, E, and F at the virology laboratory of Caen University Hospital, France. Samples analyzed were those with threshold cycle values of r-gene detection technique lower than 34. This additional characterization was performed by real-time PCR techniques using primers described in 2004 by Leruez-Ville et al. and Watzinger et al. [16, 17]. Amplifications were carried out using Hot Star Taq® DNA Polymerase kit (Qiagen, USA). A volume of 5 μL of DNA extracts were added to 20 μL of PCR mixture containing 11 μL of water, 2.5 μL of 10X buffer, 2 μL of MgCl2 at 25 mM, 1 μl of dNTPs at 10 μM, and 1 μl of enzyme. The PCR program used was 50 °C for 2 min, 95 °C for 15 min, 40 cycles at 95 °C for 15 s, and 60 °C for 1 min.

### Sequence analysis

**Fig. 2:**
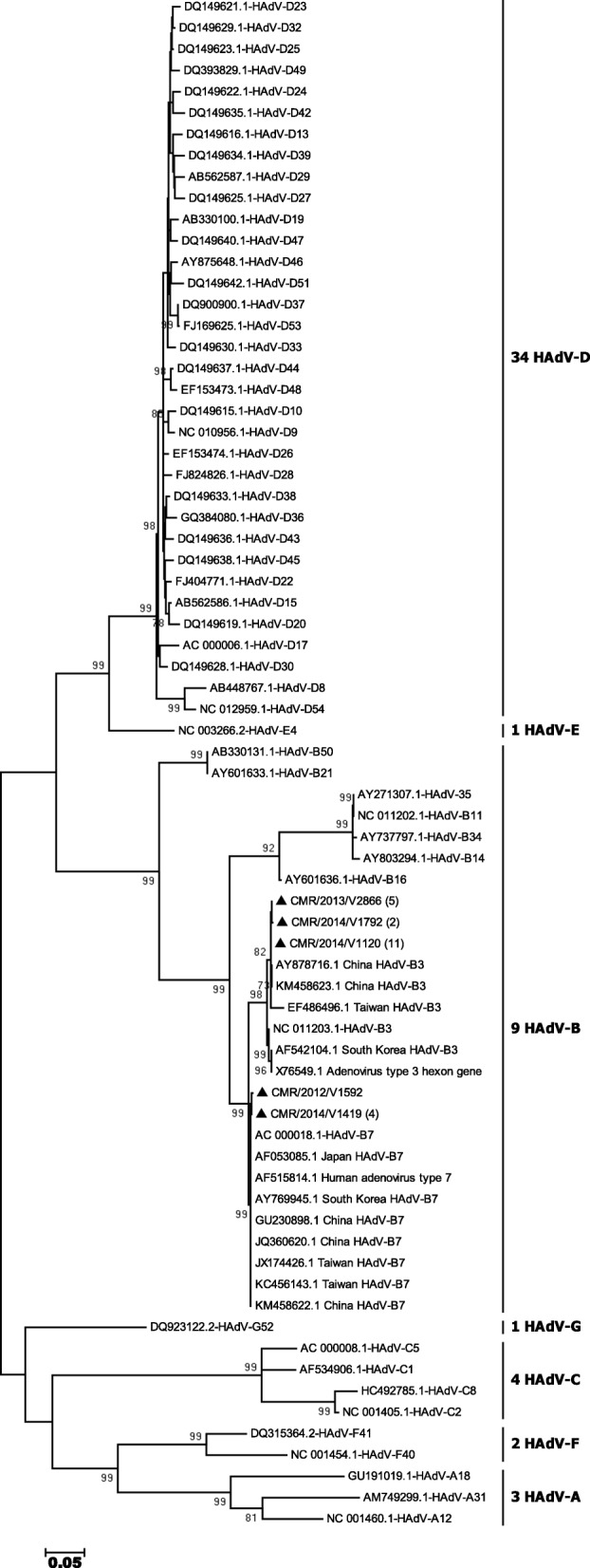
Phylogenetic analysis of partial sequences of the hexon gene (560 nucleotides) of adenovirus type 3 and type 7 collected in Cameroon from 2011 to 2014. The nucleotide sequences were aligned using the Clustal W method. The tree topology was built according to the maximum-likelihood method under the Tamura-Nei substitution model with the site heterogeneity gamma and invariant sites using the MEGA software 6. The scale bars represent the frequency of nucleotide substitutions. The accuracy of the tree was assessed by 1000 bootstrap replicates. Only Bootstrap values> 70% are presented. Cameroonian sequences are indicated by black triangles (▲) and the other sequences are the prototypes of 54 HAdV formally recognized by the ICTV. Cameroonian samples are identified in the following format: CMR (Cameroon), the year of detection and the laboratory number. The number of identical Cameroonian sequences is indicated in parenthesis following the name of the strain. Genbank accession numbers of all sequences used are shown in the tree. The scale bar represents the number of nucleotide substitutions per site between close relatives

Our sequences were cleaned and assembled using the program EDITSEQ of the software Seqman™ II Lasergene (DNA, Madison, WI, USA). Alignments were performed using the BLAST program (http://www.ncbi.nlm.nih.gov/BLAST/) for the research on Genbank of nucleotide sequences similar to the raw sequences of this study. Phylogenetic analyzes were performed using the MEGA software version 6 [18]. The gene sequences obtained in this work were aligned with reference sequences of the 54 recognized types of HAdV by the Clustal W method. We generated the phylogenetic tree using the maximum-likelihood method and Tamura Nei with the site heterogeneity gamma and invariant sites, which was the best model for our sequences. The stability of the phylogenetic tree was assessed by a 1000 bootstrap repetitions. Evolutionary distances were calculated by the Kimura-2-parameter method. The Genbank accession numbers for all sequences used in this work are included in the phylogenetic tree (Fig. 2). The nucleotide sequences of the hexon obtained in this work have been deposited in the Genbank database with accession numbers KX452120 to KX452142.

### Data analysis

Descriptive statistics such as numbers, proportions and medians were calculated to summarize the sociodemographic data. The data were also presented in tables and figures. The Chi-square test was used to assess the significance of associations between categorical variables. Associations were considered significant for *p*-value less than 5%. The data were analyzed using the R software version 3.1.0.

## Results

The gender and outpatient/inpatient ratio of recruited participants were well distributed with ratio of 1.1: 1 and 1.2: 1 respectively. All children included were under 15 years, with 63.4% having less than 2 years. The median age of these children was 19 months, IQR, (9–36) months.

### HAdV genotyping

For this study, we considered 220 HAdV positive samples. After analysis by endpoint PCR of a partial region of the hexon gene and sequencing, only 23/220 (10.5%) were amplified. The samples amplified successfully were those with low Ct (15.4 ± 4.5 vs 29.9 ± 5.9, *p* < 0.001). All 23 amplified samples were positive for HAdV-B species. The phylogenetic analysis of the 23 sequences obtained reveals that 18 of these samples are HAdV-B3 type while the remaining 5 samples are HAdV-B7 type (Fig. 2). Cameroonian HAdV-B strains clustered on the phylogenetic tree with sequences from China, Taiwan and South Korea. Of the 197 samples non-amplified by endpoint PCR, 121 were analyzed by real time PCR for screening of HAdV-A to F species. HAdV was found in 114 of the 121 analyzed samples. Six HAdV species were identified and HAdV-B (85 cases) was the predominant strain followed by 47 HAdV-C, 6 HAdV-F, 5 HAdV-E, 1 HAdV-A and 1 HAdV-D.

### HAdV-associated factors

HAdV-B was significantly more present in hospitalized patients (Table 1). The detection rate of HAdV-C was inversely proportional to the age of the children recruited in the study (*p* = 0.04). HAdV and the remaining HAdV species prevalence were not associated to age, gender, and hospitalization.

**Table 1:** Socio-demographic and Clinical factors associated with human Adenovirus species isolated in Cameroon, 2011–2014

	HAdV (231)	HAdV-A (1)	HAdV-B (108)	HAdV-C (47)	HAdV-D (1)	HAdV-E (5)	HAdV-F (6)
Age							
< 2 Years	153	1 (0,1)	61 (7,5)	31 (3,8)	1 (0,12)	4 (0,5)	6 (0,7)
2–5 Years	20	0 (0)	34 (4,2)	16 (2)	0 (0)	0 (0)	0 (0)
5–15 Years	56	0 (0)	12 (1,5)	0 (0)	0 (0)	1 (0,1)	0 (0)
NA	2	0 (0)	1 (0,1)	0 (0)	0 (0)	0 (0)	0 (0)
p-value	0,4	0,9	0,4	0,04	0,9	0,6	0,3
Gender							
Female	108	1 (0,1)	49 (6)	22 (2,7)	1 (0,12)	3 (0,4)	3 (0,4)
Male	123	0 (0)	59 (7,3)	25 (3,1)	0 (0)	2 (0,2)	3 (0,4)
*p*-value	0,9	0,3	0,7	1,0	0,3	0,6	0,9
Type							
ILI	92	1 (0,1)	31 (3,8)	20 (2,5)	0 (0)	2 (0,2)	1 (0,1)
SARI	139	0 (0)	77 (9,5)	27 (3,3)	1 (0,1)	3 (0,4)	5 (0,6)
*p*-value	0,06	0,3	< 0,001	0,7	0,4	0,8	0,2

Data are number (%), *SARI* Severe Acute Respiratory Illness, *ILI* Influenza Like Illness

### Temporal distribution HAdV-positive samples

The HAdV-B and C species were the most common and were found throughout the study period (Table 2). HAdV-E and F have noticed sporadically between 2012 and 2014. The only cases HAdV-D and A appeared in February and June 2014 respectively. Of the 23 strains that have been sequenced and analyzed, the HAdV-B3 was the predominant type and was found continuously throughout the investigation. In contrast, 5 strains of HAdV-B7 were found sporadically in 2011 and 2012.

**Table 2:** Distribution of HAdV species detected in Cameroon according to year of detection, 2011–2014

	2011	2012	2013	2014	Total
HAdV-A	0 (0)	0 (0)	0 (0)	0 (0)	1 (0,6)
HAdV-B	14 (70)	32 (69,6)	24 (58,5)	38 (63,3)	108 (64,3)
HAdV-C	6 (30)	10 (21,7)	13 (31,7)	18 (30)	47 (28)
HAdV-D	0 (0)	0 (0)	0 (0)	1 (1,7)	1 (0,6)
HAdV-E	0 (0)	1 (2,2)	2 (4,9)	2 (3,3)	5 (3)
HAdV-F	0 (0)	3 (6,5)	2 (4,9)	1 (1,7)	6 (3,6)

Data are number (%)

### Distribution of HAdV species mixed detections

HAdV species of 137/220 positive samples were obtained. Mixed HAdV interspecies detections were obtained in 30 cases (29 double infections and 1 triple infection). It is noteworthy that 22 double infections were the couple HAdV-B/HAdV-C and all HAdV-A, HAdV-D, and HAdV-F infections occur in co-detection (Table 3).

**Table 3:** Human Adenovirus interspecies codetection in Cameroun, 2011–2014

	HAdV-A	HAdV-B	HAdV-C	HAdV-D	HAdV-E	HAdV-F	Total = 137
HAdV-A	0	1	0	0	0	0	1 (0.7)
HAdV-B	–	83	22	0	0	1	108 (78,8)
HAdV-C	–	–	23	0	0	1	47 (34,3)
HAdV-D	–	–	–	0	0	0	1 (0,7)
HAdV-E	–	–	–	–	1	4	5 (3,6)
HAdV-F	–	–	–	–	–	0	6 (4,4)
Mono	O	83	23	0	1	0	107 (78,1)
Double	1	24	23	0	4	6	29 (21,2)
Triple	0	1	1	1	0	0	1 (0,7)

Data are number (% = number/137), Monoinfection (Diagonal) or Coinfection (Matrix)

### Phylogenetic analysis

A similarity rate of 99.9 to 100% was obtained when comparing the 861 nucleotides and deduced amino acid of the Cameroonian hexon gene sequences to reference strains B3 (NC_011203.1) and B7 (AC_000018.1). In comparison with the B7 reference strain, B7 sequences of this study have only two variable residues in all sequences from Cameroon. Eight variable positions were observed with respect to the Cameroonian B3 sequences and B3 prototype strain.

## Discussion

This study reports the different HAdV species involved in respiratory infections in Yaounde, Cameroon between September 2011 and July 2014. To the best of the information available to date, this is the first study that performed a molecular characterization of HAdV strains isolated from patients with acute respiratory infections in Cameroon. Only 23 samples positives in generic quantitative PCR could be amplified by typing PCR on the hexon gene during this work. This low rate of amplification may mainly be due to the significantly low viral load of unamplified samples (mean Ct unamplified samples = 28.9 +/− 5.9 vs. 15.4 +/− 4, 5 for amplified samples, *p* < 0.001). As nested PCR is known to be more sensitive than PCR, it would have been possible to amplify and sequencing a larger number of positive samples if nested PCR had been used. Out of the 137 HAdV positive samples that were characterized, 78.5% (108) were virus HAdV-B species and 34.8% (47) HAdV-C. In fact, these species represent the most common species identified worldwide in acute respiratory infections [15, 19–27]. In contrast to this study, where HAdV-B was the predominantly species, two other African studies conducted in Egypt and Senegal have showed the predominance of the HAdV-C species [22, 26]. This could be partially explained by the inclusion of only patients with ILI in these studies, contrary to this work which also recruited patients with severe acute respiratory infections. In fact, the HAdV-B species was significantly predominant in hospitalized patients during this study (*p* < 0.001). Another study in Chile from 1988 to 1996 had also shown that HAdV-B infections were more severe than HAdV-C infections [27]. Previous studies have shown that HAdV species A (31) and F (40 and 41) were commonly found in the gastrointestinal tract [28, 29]. Similarly, HAdV-D (8, 19 and 37) are found in ocular system [29–32]. Similar to other authors [19, 33], we detected these species HAdV in respiratory samples, showing that their tropism can include respiratory tract. Unlike previous studies [22, 24], during this work 21.9% (30/137) of interspecies HAdV mixed infections were recorded. Nevertheless, this interspecies coinfection frequency remains similar to that (19%) recorded by Wang et al., in 2013 in Taiwan [25]. The comparison of the partial sequence of the hexon obtained for the Cameroonian HAdV-7 and HAdV-3 types and the reference strains reveals a very low rate of divergence. This result confirms the high stability of the HAdV genome also reported in previous studies [34]. The principal limitation of the present study is the low rate of amplification of HAdV-positive samples by end point PCR.

## Conclusion

The various HAdV species involved in acute respiratory infections in Cameroon are showed in this study. HAdV-B and C were the predominant species and HAdV-B was associated to hospitalization. These results will form the basis for the implementation of the work to determine the burden of HAdV infection in Cameron.

## Abbreviations

HAdV: Human adenovirus
